# Antenna Design and SAR Analysis on Human Head Phantom Simulation for Future Clinical Applications

**DOI:** 10.4236/jbise.2017.109032

**Published:** 2017-09-12

**Authors:** Felipe Pablo Perez, Joseph Paul Bandeira, Jorge J. Morisaki, Seshasai Vamsi Krishna Peddinti, Paul Salama, James Rizkalla, Maher E. Rizkalla

**Affiliations:** 1 Department of Medicine, Geriatric Medicine Section, Indiana University School of Medicine, Indianapolis, USA; 2 Department of Bio-Engineering, University of Illinois at Chicago, Chicago, USA; 3 Department of Electrical and Computer Engineering, Indiana University Purdue University Indianapolis (IUPUI), Indianapolis, USA; 4 Baylor University Medical Center, Dallas, USA

**Keywords:** Antenna, Neuro, Alzheimer Disease, Diagnosis, COMSOL, SAR

## Abstract

**Background:**

The rapid development of a variety of devices that emit Radiofrequency Electromagnetic fields (RF-EMF) has sparked growing interest in their interaction with biological systems and the beneficial effects on human health. As a result, investigations have been driven by the potential for therapeutic applications, as well as concern for any possible negative health implications of these EM energies [1-4]. Recent results have indicated specific tuning of experimental and clinical RF exposure may lead to their clinical application toward beneficial health outcomes [5].

**Method:**

In the current study, a mathematical and computer simulation model to analyze a specific RF-EMF exposure on a human head model was developed. Impetus for this research was derived from results of our previous experiments which revealed that Repeated Electromagnetic Field Stimulation (REMFS) decreased the toxic levels of beta amyloid (A*β*) in neuronal cells, thereby suggesting a new potential therapeutic strategy for the treatment of Alzheimer's disease (AD). Throughout development of the proposed device, experimental variables such as the EM frequency range, specific absorption rate (SAR), penetration depth, and innate properties of different tissues have been carefully considered.

**Results:**

RF-EMF exposure to the human head phantom was performed utilizing a Yagi-Uda antenna type possessing high gain (in the order of 10 dbs) at a frequency of 64 MHz and SAR of 0.6 W/Kg. In order to maximize the EM power transmission in one direction, directors were placed in front of the driven element and reflectors were placed behind the driven element. So as to strategically direct the EM field into the center of the brain tissue, while providing field linearity, our analysis considered the field distribution for one versus four antennas. Within the provided dimensions of a typical human brain, results of the Bioheat equation within COMSOL Multiphysics version 5.2a software demonstrated less than a 1 m˚K increase from the absorbed EM power.

## 1. Introduction

Although prior long-term studies in AD mouse model demonstrated that REMFS reversed cognitive impairment by decreasing A*β* levels with no significant side effects [6], extrapolation of data that would allow for safe implementation in humans has been limited by the increased potential risk of thermal injury, differences in penetration, and a lack of consensus on the precise mechanisms involved. Together, these conditions have created a critical gap between our understanding of the mechanism of this beneficial effect, and the precise human REMF exposure settings to achieve it. It was precisely the desire to bridge this gap that prompted our initial research to determine the EMF frequencies considered suitable for human exposures. Our experiments determined that RF-EMF at 64 MHz was most appropriate for human studies. Four important characteristics supported this frequency. First, it is a non-thermal and non-ionizing energy, and therefore less likely to cause cellular damage. Secondly, 64 MHz is used by MRI systems, and affords safe exposure conditions. Thirdly, 64 MHz is comparable to our previous studies utilizing 50 MHz in which we demonstrated an enhancement of several AD-related pathways, including the involvement of cytoprotection in human lymphocytes via augmentation of the HSF1 pathway [7]. Lastly, it is close to the frequency of 75 MHz, which is the known resonant frequency of the human body [8], at which the organism absorbs up to ten times as much power as when it is not in resonance [9-11]. As a result, more power is absorbed, and the amount of energy to obtain the minimum SAR required for biological responses is significantly less.

Recently, our ongoing research has demonstrated that REMFS significantly decreased the levels of the A*β*, the most likely cause of Alzheimer's disease (AD), in human neuronal cell cultures. The study involved the application of REMFS at 64 MHz with a SAR of 0.6 W/Kg daily during one hour for 21 days. Treatment results revealed a 58.35% decrease in toxic A*β* levels compared to non-treated human primary neuronal cultures. Notably, all results were achieved with no signs of cellular toxicity in treated cultures [12]. Based on the results of our work with human neurons, and studies performed by other researchers in Alzheimer's disease mouse models [13], we hypothesize that REMFS is a new non-invasive therapeutic strategy to treat AD in human.

However, before clinical trials can be considered, it is important to understand that the mouse model results cannot easily be extrapolated to humans. Reason being, the mouse's physiological characteristics differ significantly from that of a human, and exposure to the same incident fields would result in quite different internal fields. This is because the energy absorbed is directly related to the internal fields, which are the electromagnetic fields inside the object, and not the electromagnetic fields incident upon the object. Hence, it is important to determine what external fields would produce similar internal fields inside both mouse and man. Another difference to consider is tissue penetration, which is inversely proportional to frequency. For instance, the cell phone frequency of 918 MHz reaches a penetration depth of 4 cm into a human skull. Whereas, such a depth would be considered whole-body exposure for a mouse. Also, considering the mean human head thickness is 19.4 cm, a wave with a penetration of at least 9.7 cm would be required to irradiate the center of a human head, making the 4 cm depth of a cell phone's frequency ineffective [14-16].

Therefore, prior to human testing, computer simulation validation is necessary to adjust EMF parameters so that similarities in dosimetry between cell cultures, animal exposure, and human exposure are found.

The current study proposes to develop a mathematical model and utilize computer simulations to design an antenna that will produce a SAR of 0.6 W/kg (the energy needed for biological effect with uniform distribution throughout the human brain). To this end, we will utilize validated simulation programs (COMSOL Multiphysics version 5.2a) to design the antenna dimensions and to determine precise parameters of the exposure, such as the power, distance from the brain, etc. We will also perform simulations to analyze and perform simulations for the SAR, temperature, electric field, and magnetic field, all of which will aid in the construction and design of an antenna for the purpose of irradiating a human brain phantom with similar anatomical geometry, size, and dielectric properties as that of the human brain.

It is our hope that this groundbreaking research will pave a road away from the current pharmacology-dependent paradigm, and guide us toward a new understanding of an electromagnetic-biological system interaction aimed at the treatment of Alzheimer's disease.

## 2. Method

The model was approached by solving both Bioheat and EM equations with the proper boundary conditions. COMSOL Multiphysics version 5.2a software was used to combine these equations in order to determine the electric field and the SAR values within the brain tissue. The bone/skull boundary conditions were introduced by matching the tangential E and H fields at the boundary. The reflections and transmissions patterns were determined for the simulation involving a 16 cm size head, and for a 6.5 mm skull material surrounding the brain tissue. The wave equation taken under a frequency domain in the simulation is written as:

(1) $$\nabla \times \mu_{r}^{-1}(\nabla \times E)-k_{0}^{2}({ɛ}_{r}-\frac{j\sigma }{\omega {ɛ}_{0}})E=0$$

where *μ_r_* is the relative permeability of the neuron cells, *ε_r_* is relative permittivity of the neuron cells, σ is the conductivity of the material, *ε*_0_ is the absolute permittivity, and *ω* is the radian frequency. The model inputs were taken as 293.15 K for temperature and 1 atm for absolute pressure. The antennae were given a perfect electric conductor boundary condition, which was given by:

(2) n×E=0

The lumped port boundary condition was given to the other end of the antennae. Each antenna has one lumped port, which follows the equation,

(3) $$z=\frac{V_{1}}{I_{1}}$$

The height and width of the lumped port was taken as 0.2 m and 0.01 m respectively. The characteristic impedance was taken as 50 ohms.

For the Bioheat Transfer conditions, the ambient temperature was taken 293.15 K, ambient absolute pressure was 1 atm, and ambient solar irradiance was 1000 W/m^2^ with zero ambient relative humidity and wind velocity. The following are the equations for biological tissue condition,

(4) $$\rho C_{p}u\cdot \nabla T+\nabla \cdot q=Q+Q_{\text{bio}}$$

(5) q=−k∇T

where *ρ* is the density of the material, *C_p_* is is the heat capacity at constant pressure, and *k* is the Thermal conductivity.

The Bioheat condition has the following equations,

(6) $$\rho C_{p}u\cdot \nabla T+\nabla \cdot q=Q+Q_{\text{bio}}$$

(7) $$Q_{\text{bio}}=\rho_{b}C_{pb}\omega_{b}(T_{b}-T)+Q_{\text{met}}$$

where *T_b_* is the arterial blood temperature, *C_pb_* is the specific heat of blood, *ω_b_* is the blood perfusion rate, *ρ_b_* is the density of the blood, and *Q_met_* is the metabolic heat source. The brain and/or the neurons have a thermal insulation which follows the equation,

(8) −n·q=0

where *q* is heat flow rate.

Finally, the brain and/or neurons cells are selected as the electromagnetic heat source, this follow the equations,

(9) $$\rho C_{p}u\cdot \nabla T=\nabla \cdot (k\nabla T)+Q_{e}$$

(10) $$Q_{e}=Q_{\text{rh}}+Q_{\text{ml}}$$

(11) $$Q_{\text{rh}}=\frac{1}{2}\text{Re}(J\cdot E^{*})$$

(12) $$Q_{\text{ml}}=\frac{1}{2}\text{Re}(i\omega B\cdot H^{*})$$

where *H* is the magnetic field, *B* is the magnetic flux density, *Q_e_* is heat equilibrium, *Q_rh_* is heat addition, and *Q_ml_* is the microlayer evaporation heat.

## 3. Results and Discussions

The results obtained from the human brain phantom design simulation using COMSOL Multiphysics version 5.2a help to understand the impact of the antennae on the skull, brain, and neurons. A practical design may consist of coaxial cables connected to the antennae at the lumped ports. Results from four antennae and a single antenna placed around a simulated brain were obtained. The simulation was run using acceptable dielectric values of brain, bone, and the neurons. All simulations were run on 64 MHz frequency. The electric field measured was presented in a volumetric plot to aid the analysis of the E field in all the dimensions. It should be noted that the E field obtained is normalized, and can be called emw.Qrh. This file within COMSOL estimates the normalized value of the electric field, *E.* To obtain the SAR values, the following expression was used:

$$\log_{10}(\sigma_{\text{neuron}}*\text{emw}.\text{Qrh}/\rho_{\text{neuron}}).$$

where σ_neuron_ is the relative conductivity of the neuron, emw.Qrh is a parameter given by COMSOL for the normalized *E* field value, and *ρ*_neuron_ is the mass density of the neuron. It should be noted that both the *E* field and SAR are obtained in log scale.

**Figure 1:**
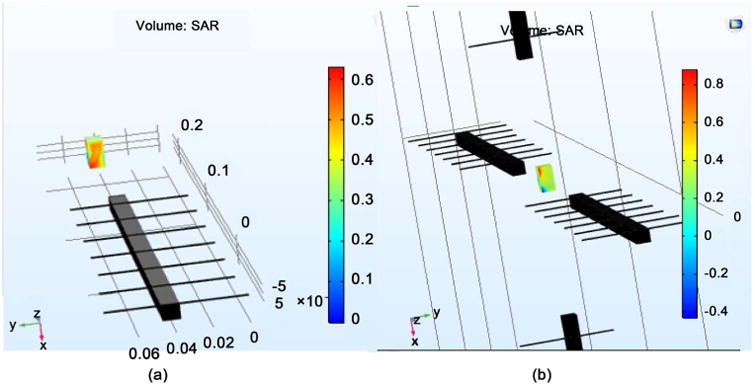
Volumetric plot for SAR measurement for (a) single antenna; (b) 4 antennae.

**Figure 2:**
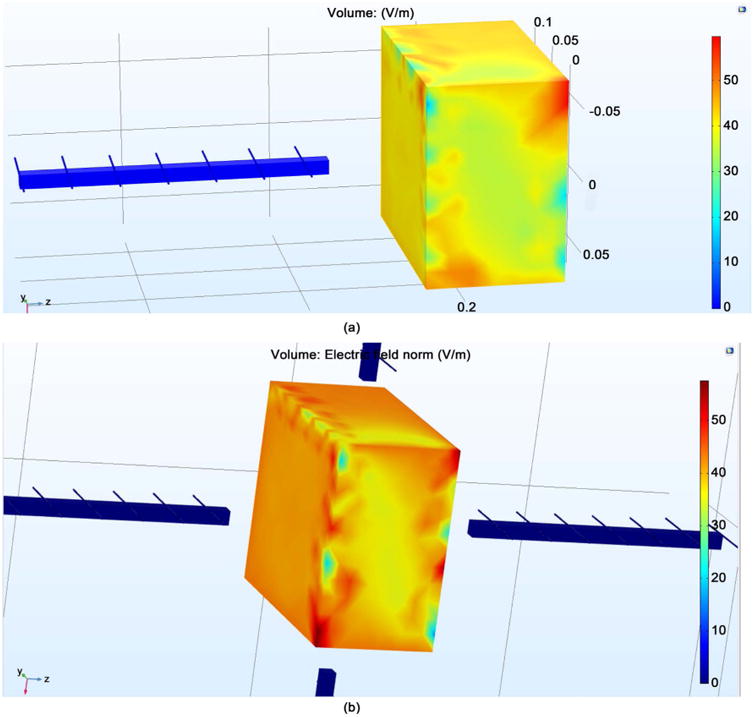
The E-Field distribution for (a) single antenna; (b) four antennae.

**Figure 3:**
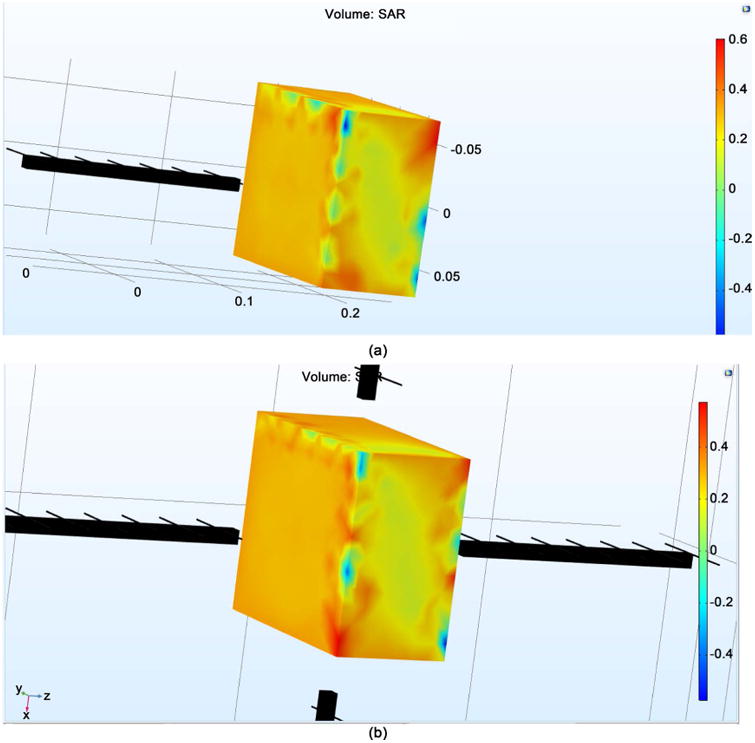
The SAR distribution from (a) single antenna; (b) four antennae.

Figure 1 shows the SAR on a single neuron utilizing both a single antenna and four antennae at 64 MHz frequency. To achieve a SAR of 0.6 W/Kg, a single antenna requires approximately 34 V/m of E field. Whereas, four antennae require approximately 24.5 V/m of E field. It can be seen under both conditions that the SAR value is not uniform throughout. This is attributed to the E propagation, which gives higher E value at the surface interface while giving lower values when far from the surface. The voltages obtained are taken as threshold values to obtain the required SAR for single antenna and four antennae, respectively. Since the penetration depth is identical in all four radiating antennae, it is clearly demonstrated that the single antenna displays better linearity inside the test parameters. Figure 2 gives the Electric Field on the brain model via EM wave simulation when a single antenna and four antennae were used. The outer layer, representing the brain, has approximately 45 V/m when the first case uses 34 V/m while the second case uses 24.5 V/m directed towards the surface of the brain. It can be seen that the surface of the brain has a uniform E field, with uniform field distribution in all directions. The E field obtained from the Figure 2 gives values close to the desired SAR of 0.6 W/Kg. The SAR distribution for both one antenna and four antenna systems is given in Figure 3. The SAR value was near 0.55 W/kg. There is greater uniform distribution of SAR in the four antennae system than that obtained from the neuron simulation within the four antennae system. This is attributed to the symmetrical penetration depths of the four antennae within the four the directions. It may be concluded that that neurons at any location inside the brain can be impacted by the E field supplied in all directions.

**Figure 4:**
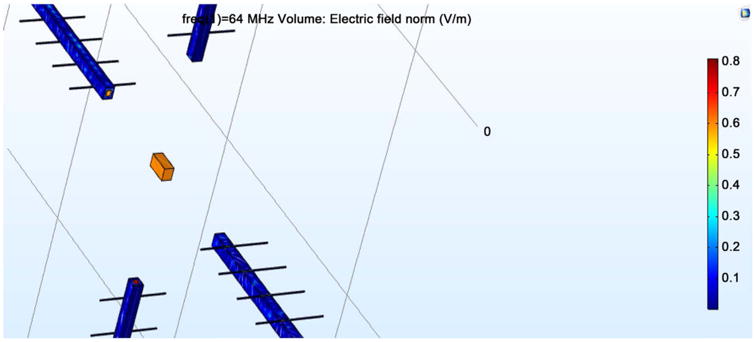
Propagation of E field.

Figure 4 shows the E field radiating from the transmission line. This field is sent to the neuron via the bone material. The transmissions lines within the antennae utilize copper as the material on the outer layers of the antenna, and are the primary source of field emission.

**Figure 5:**
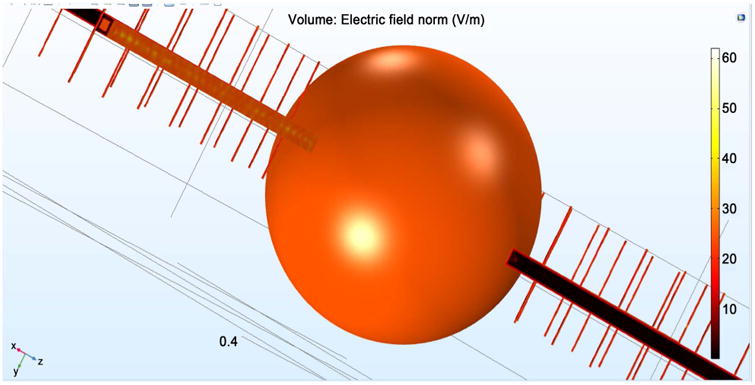
E field for a human skull surrounding the brain tissue.

Figure 5 illustrates the field distribution directed at the skull surrounding the brain tissue. Figure 5 shows the E field distribution for the given EM strengths that were directed at the brain without the surrounding bone. The bone, which is skull in this particular application has been given a spherical geometry, so as to closely resemble the human cranium. The inner sphere simulated brain tissue, and the outer layer simulated bone tissue, and gives approximately 24.5 V/m as its E field. This is the same value obtained in the case of neuron exposure with the four antennae system. Importantly, it can be seen that a nearly 30% drop in field strength occurred inside the brain tissue in response to the simulated bone tissue. This indicates that the original EM power should be increased by 30% in order to satisfy the SAR requirement for uniform brain penetration.

## 4. Temperature Impact

**Figure 6:**
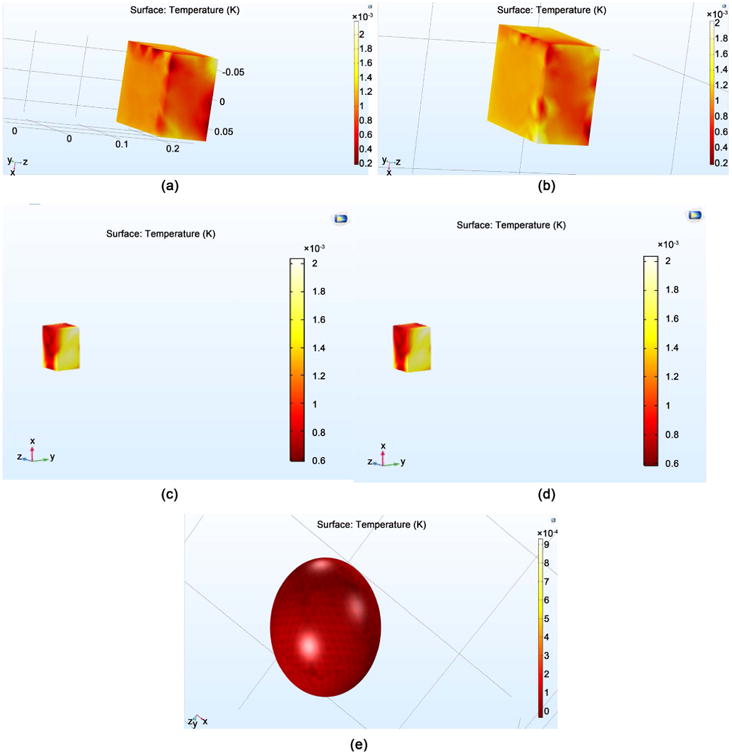
The temperature distribution for (a) The surface temperature for one antenna system; (b) The surface temperature for four antenna system (c) The penetration depth for one antenna; (d) The penetration depth from four antenna system; (e) The skull surrounding the brain tissue.

As mentioned earlier, the temperature distribution within the simulated brain tissue is important so as to guarantee no rise in brain temperature secondary to the application of EM power. Figure 6 gives the temperature distributions on the surface with one antenna system (a) and four antenna system (b), and a view displaying the temperature rise due to EM penetration for the one antenna system (c), and four antenna system (d). Of note, the temperature of the bone surrounding the brain is also provided.

## 5. Conclusions

A growing body of experimental work is emerging on the interaction between EMF and biological systems, and through the use of computer simulations its users can now visualize the effects of EM fields of defined frequency, intensity, and SAR on selected tissues and organs. Our simulation results display the response of a brain model and surrounding bone to a predetermined (64 MHz frequency) electromagnetic field in terms of power absorbed, temperature change, and distribution as represented by SAR.

The data presented herein suggest minimal EMF reflection of fields beyond the predetermined boundaries. Important electrical properties of brain tissue within a (14 cm × 16 cm × 9 cm) typical human brain model have been investigated, and brain cells interfaced with skull/bone tissue of 6.5 mm thickness surrounding a 16 cm diameter of phantom human head have been taken into account. We determined that to achieve the therapeutic SAR of 0.6 W/Kg, a single antenna requires approximately 34 V/m of E field. Whereas, four antennae require approximately 24.5 V/m of E field. Furthermore, we discovered that a 30% reduction in field penetration secondary to bone/skull could be offset by a 30% increase in the initial power to allow for a uniform SAR of 0.6 W/Kg in the human head phantom without causing an increase in temperature. When compared to the 1-antenna system, the 4-antenna system achieved a field distribution throughout the brain model that was slightly better, although not significant.

The proposed practical antenna model could be constructed within reasonable size parameters. The Yagi-Uda layout with driven element would have a length lambda/64 = 0.0732 m. One reflector would be of length 0.0769 m (lambda/64% + 5%) and spacing 0.0293 m (0.2 * lambda/32). The five directors would be of length 0.0696 m (lambda/64% − 5%) and spacing 0.0293 m (0.2 * lambda/32). All elements would have diameter 0.0012 m (0.0085 * lambda/32) with maximum dimensions of about 0.08 m and 0.18 m.

Although our results suggest plausibility for the development of a device that could provide a uniform SAR with beneficial biological effects that would include a decrease of toxic A*β* levels and improvement of memory in AD human clinical trials, the REMFS research parameters determined in this study will need to be applied to the Specific Anthropomorphic Mannequin (SAM) in order to validate our computer simulations before moving on to clinical trials. This is reserved for future considerations. Importantly, the forthcoming results of this study may have useful application toward cellular communications, microwave devices, power lines, and EM therapeutic procedures.

It can be expected that the current study may face some challenges in the practical model related to variability of human head size and tissue properties, as well as inclusion of other layers such as muscles and fluids inside the brain. Additionally, the complex nonlinearity of the brain tissue may be another factor of requiring further elaboration. These also are reserved for future considerations.
